# Search for axions and dark photons using single molecule magnets

**DOI:** 10.1038/s41598-026-66492-3

**Published:** 2026-09-07

**Authors:** Jose R. Alves, Manfred Lindner, Farinaldo S. Queiroz, Manoel S. Vasconcelos

**Affiliations:** 1 Departamento de Física, Universidade Federal do Rio Grande do Norte, Natal, RN 59078-970 Brasil; 2 International Institute of Physics, Universidade Federal do Rio Grande do Norte, Campus Universitário, Lagoa Nova, Natal, RN 59078-970 Brazil; 3 Max Planck Institut fur Kernphysik, Heidelberg, Germany; 4 Millennium Institute for Subatomic Physics at the High-Energy Frontier (SAPHIR) of ANID, Fernandez Concha 700, Santiago, Chile; 5 Departamento de Física, Facultad de Ciencias, Universidad de La Serena, Avenida Cisternas 1200, La Serena, Chile; 6 Programa de Pós Graduação em Física, Universidade do Estado do Rio Grande do Norte, Mossoró, RN 59610-210 Brasil

**Keywords:** Chemistry, Physics

## Abstract

Molecular magnets, although analogous to familiar macroscopic magnets, offer a platform for next generation magnetic storage technologies with far higher data densities and prospective applications in quantum information science. When exposed to an external magnetic field, single molecule magnets enter a metastable configuration that is exceptionally sensitive to low energy excitations. Energy deposited by a dark matter particle can trigger the relaxation of a metastable molecule, releasing Zeeman energy that subsequently propagates through neighboring molecules. This magnetic avalanche encodes the energy deposited in the initial excitation. By combining concepts from chemistry, condensed matter physics, and particle physics, we show that dysprosium and manganese molecules can achieve more than an order of magnitude improvement in sensitivity to dark photon and QCD axion models, respectively, compared with existing detection methods.

## Introduction

**Fig. 1:**
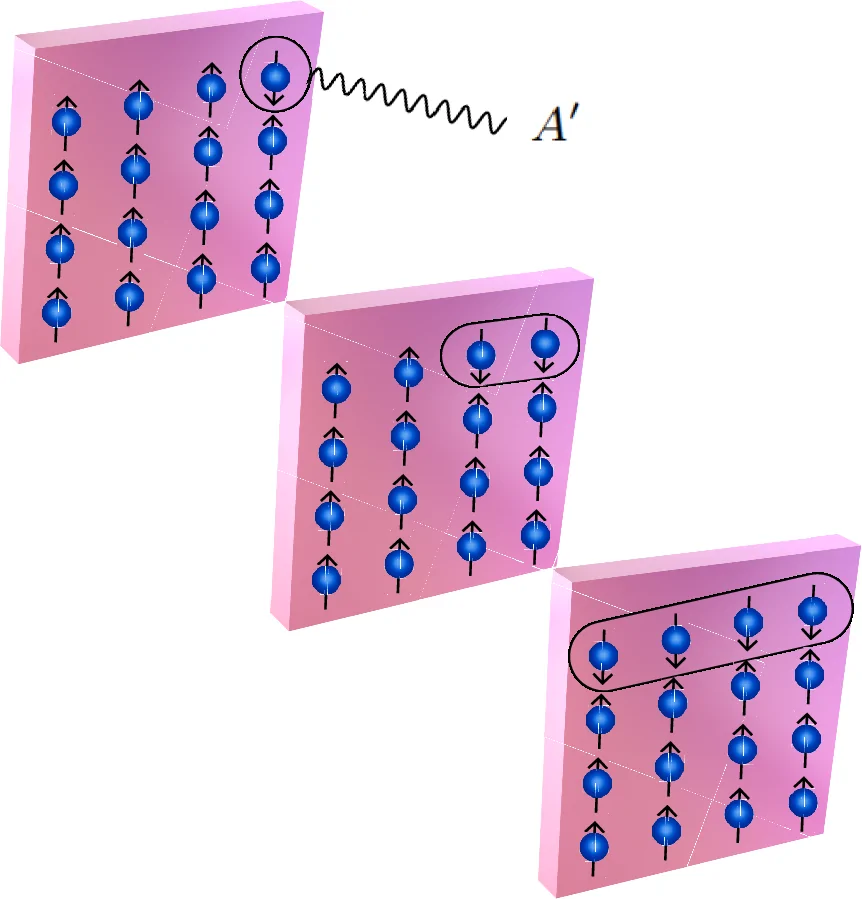
Sketch of the detection mechanism proposed in the paper. From left to right: the DM particle hits one of the molecules in the metastable state, which relaxes, releasing the Zeeman energy stored, which then propagates to the neighboring molecules, as shown by the red dashed circle increasing in size, in a process known as magnetic avalanche.

The existence of non-baryonic dark matter (DM), a dominant component shaping galactic structures and galaxy clusters, is firmly established through extensive cosmological and astrophysical evidence^6^. Yet its fundamental nature remains elusive. The two leading interpretations—primordial black holes^7^ and elementary particles—present distinct pathways; here we focus on the latter. This approach necessitates non-negligible interactions between DM and Standard Model (SM) particles to enable experimental detection (Fig. 1).

Searches for DM via nuclear scattering^8–10^, astrophysical signatures^11,12^, and accelerator production^13,14^ have excluded many minimal weakly interacting DM models. Future experiments like LUX-ZEPLIN^15^, XENONnT, and PandaX-4T may soon achieve detection. However, for sub-GeV DM masses, nuclear recoils are suppressed. A DM particle with mass *M* will carry energy $E=1/2 M v^2 \sim 10^{-6}M$, yielding recoils below the keV thresholds of conventional detectors. While electron-recoil experiments extend sensitivity to MeV masses^16,17^, lighter DM remains inaccessible, motivating our novel approach.

Notably, dark matter particles in the Milky Way halo may undergo acceleration through elastic collisions with high-energy galactic cosmic rays and astrophysical neutrinos^18–23^. This inevitable “boosted dark matter” (BDM) component, though small, carries sufficient kinetic energy to be constrained by direct detection experiments. However, when the mediator of the DM-target interaction is light, ($M_{med}\ll q$), sensitivity degrades due to momentum-dependent form factors. While existing BDM studies focus primarily on keV-scale masses, we target the unexplored sub-eV regime, where conventional detectors lack sensitivity.

**Fig. 2:**
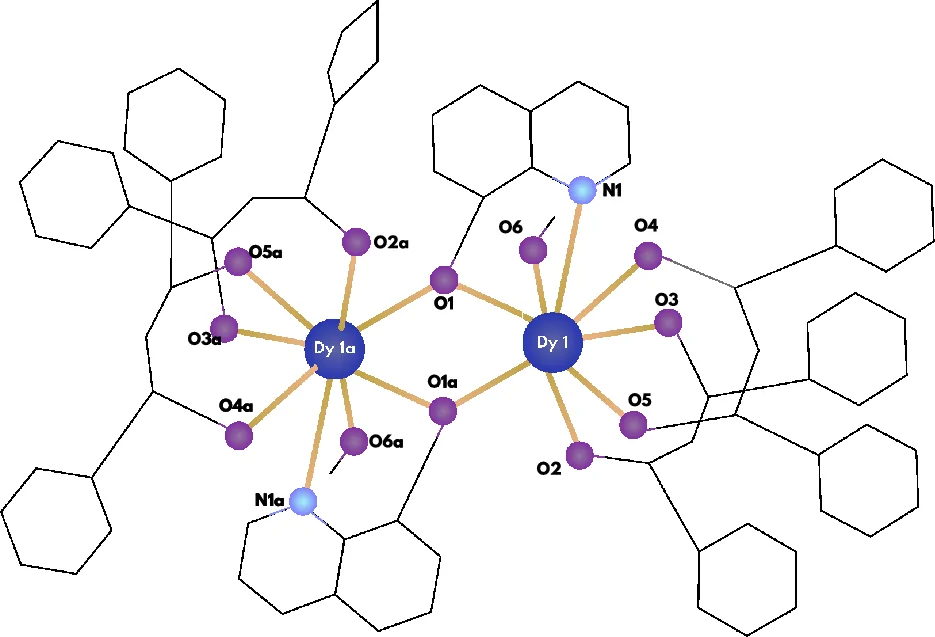
It illustrates the dinuclear dysprosium. The dark blue spheres represent the dysprosium nuclei, the purple circles the oxygen atoms, and the light blue spheres the nitrogen.

**Fig. 3:**
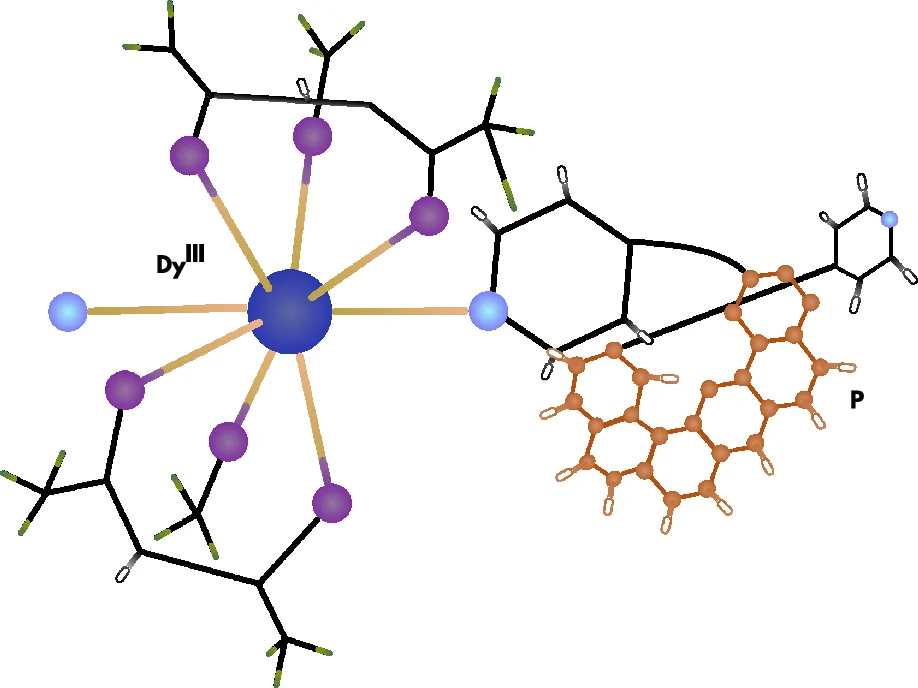
Figure displaying Dy$^{\text {III}}$. The dysprosium atom is in dark blue, oxygen in purple, and nitrogen in light blue.

Motivated by this gap, we leverage condensed-matter systems—building on proposals using semiconductors^24,25^, photonic nanostructures^26^, Fermi-degenerate materials^27^, and magnetic bubbles^5^. Specifically, we propose dysprosium-based single-molecule magnets (SMMs) as BDM detectors: the dinuclear complex Dy$_2$, and the complex Dy$^{\text {III}}$ (illustrated in Figs. 2, 3) both exhibiting robust SMM behavior^1,2^. These molecules are described as effective spin systems, where we will denote here *S* as the effective spin for brevity. We further incorporate far-infrared spectra of Manganese, $Mn_{12}$-acetate^3,4^ to probe *axion* masses across complementary frequency bands.

**Fig. 4:**
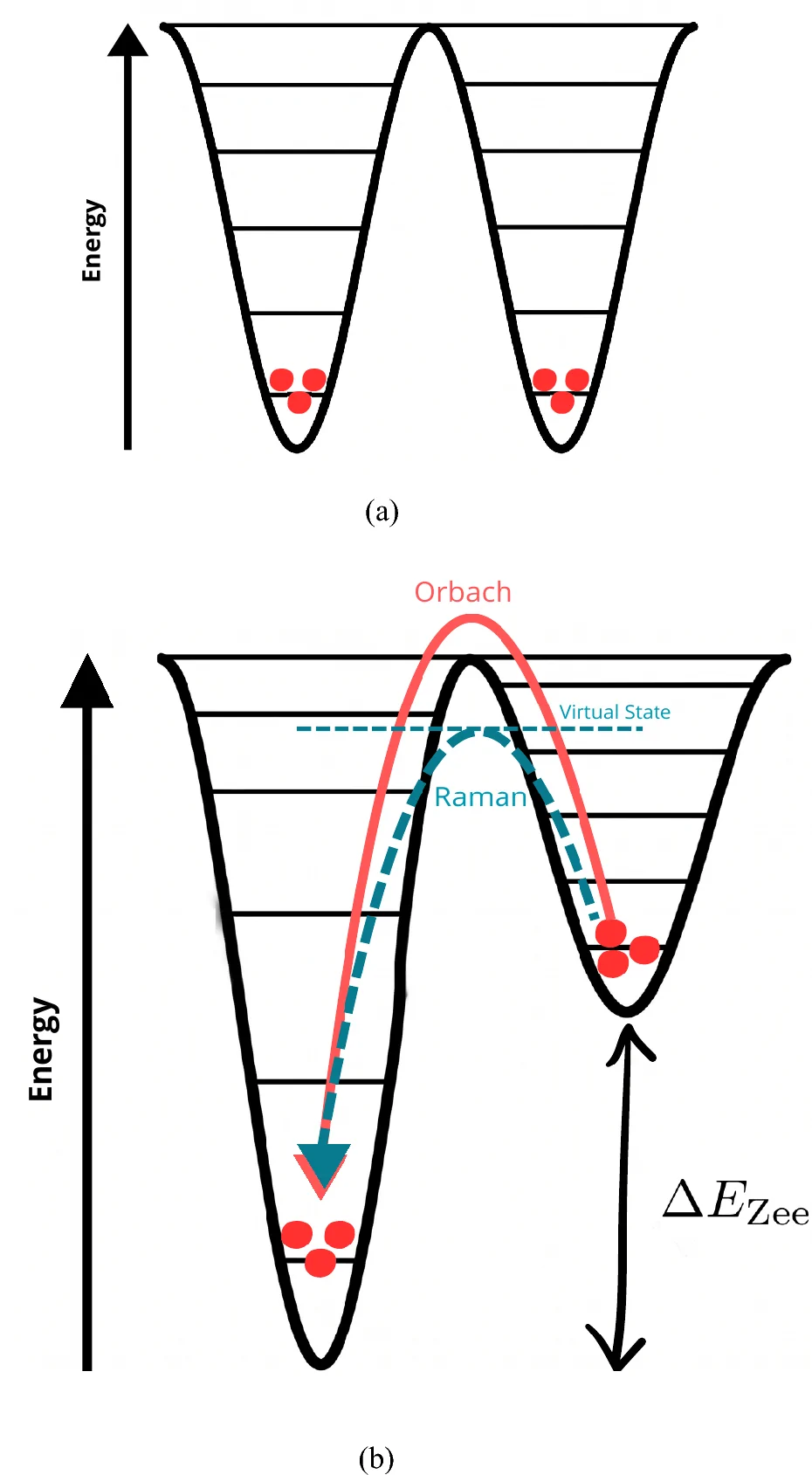
The potential felt by an SMM molecule, (**a**) shows the potential without an external magnetic field, picturing the degenerate vacuum, while (**b**) pictures one of the vacuum lifted due to the presence of an external magnetic field. The mechanism proposed focuses on the latter.

These systems maintain metastable magnetic states (See. Fig.4a–b) for extended periods at cryogenic temperatures *T∼ 0.1* K. They have attracted considerable interest due to their applications in magnetic storage and potential use as qubits in quantum computing^28^. This characteristic of staying in a false vacuum with a relatively long period allows us to probe dark sectors via the absorption of a BDM as illustrated in Fig. 1.

We derive the physics reach of this new method for two popular dark matter models: *dark photon* and *axion*. The *dark photon* arises as the gauge boson of an extended *U(1)'* symmetry in the SM Lagrangian via the interaction term,^29^,

1 $$\begin{aligned} \mathcal {L}_{\text {DP}} \supset - \frac{\varepsilon }{2}F_{\mu \nu }F'^{\mu \nu }, \end{aligned}$$

where *ε * is the kinetic mixing. The *axion* particle has been introduced to solve the strong CP problem as a result of the Peccei–Quinn symmetry^30–32^. In the end, this solution induces a coupling to the electromagnetic field strength tensor described by^33,34^,

2 $$\begin{aligned} \mathcal {L}_{\text {a}} \supset -\frac{1}{4}g_{a\gamma \gamma } a F_{\mu \nu } \tilde{F}^{\mu \nu }. \end{aligned}$$

Because this work spans several disciplines, we begin by introducing the essential concepts of SMMs and molecular absorption. We then show that, for particle masses below 1 eV, these systems provide a uniquely powerful platform where particle physics, condensed matter physics, and chemistry intersect, enabling a detection strategy with the potential to surpass the sensitivity of existing experimental approaches by more than one order of magnitude.

## Single molecule magnets

In this section, we outline the key properties of SMMs that underpin their use as dark-matter sensors and review the essential principles governing absorption in SMMs to establish how the ensuing magnetic avalanche encodes the energy deposited by an incident DM particle.

**Fig. 5:**
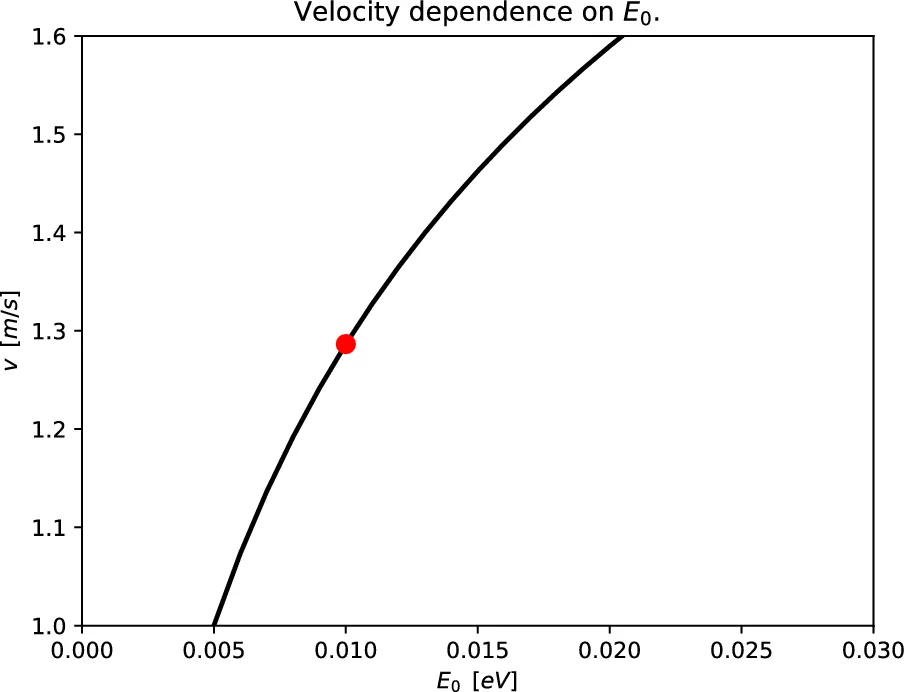
Velocity dependence on the deposited energy based on Eq. (5). The behavior is as expected: the greater the energy deposited, the higher the velocity. The red dot shows the expected velocity for an energy deposition around $10^{-2}$eV. This plot is calculated for a Zeeman energy of $10^{-4}$eV for an anisotropic energy barrier of *U = 100* K. The velocity also depends on how much Zeeman energy is stored, which means that the general behavior will change by changing the temperature at which the sample is kept.

SMMs exhibit magnetic properties similar to those of bulk magnetic material. The term *single-molecule* magnet means that the considered molecule is a magnet itself, with a unique domain. In bulk magnets, the magnetic moment arises from the presence of unpaired electrons, meaning their spins form parallel/antiparallel arrangements within domains (this is what we call the areas separated by Bloch domain walls). As well as in bulk materials, the SMMs can be magnetized by an external magnetic field, and are demagnetized when this external magnetic field is removed. We work with lanthanides, which are a class of f-block metals, in order to describe its magnetic properties, we need to consider spin–orbit coupling terms, characterized by the quantum number J, i.e., the total angular moment, and their splitting by the applied field, leading to sublevels that are described by $E_J$ instead of quantum numbers $E_S$^35^.

It has been found that its magnetization slowly relaxes with the removal of an applied external magnetic field, and the relaxation timescale is different from that of bulk materials; however, some molecules can have their relaxation timescale made large enough for the proposed experiment for low temperatures, as in Eq. (4). The relaxation time for this process will be discussed below. Therefore, these new molecules have induced a new concept in the field of magnets, generating interesting experimental and theoretical consequences, such as out-of-phase signals in AC studies of magnetic properties^36^. Most of these molecules exhibit a large spin ground state *S* that may appear due to exchange of ions in the magnetic core (as is the case for Mn12-acetate, whose S is equal to 10^35,37^) or competing antiferromagnetic interaction between the spins of the ions, alongside an axial zero-field splitting which separates the *(2S + 1)* $m_s$ projections of the spin in the *z*-axis (or easy axis of the molecule). This splitting is characterized by a parameter *D* present in the Hamiltonian that describes these molecules, which is given by,

3 $$\begin{aligned} H \supset D S_z^2. \end{aligned}$$

This Hamiltonian nicely describes the system at low temperatures, because for *D < 0* the low-lying energy levels are those with the highest $|E_S|$ value, in this case $E_S = \pm S$. The magnetization of the molecule is associated with each of the $E_S = \pm S$ sublevels having a particular orientation along the axial anisotropy axis; thus, $E_S = +S$ corresponds to spin up, whereas $E_S = -S$ corresponds to spin down. A pictorial view of the splitting with and without an applied external magnetic field can be found in Fig. 4.

In Fig. 4a, we plot the crossover between the energy levels and a double well, including a barrier of size *U*, indicating on the left the negative $E_S$ levels and on the right side, the positive $E_S$, just to illustrate the degeneracy of the eigenstates, without an applied external magnetic field. In Fig. 4b, we exhibit the potential in the presence of an external magnetic field, showing a metastable vacuum state with energy equal to $2\mu _B\, g\, J\, B$ with respect to the true vacuum, where $\mu _B$ is the Bohr magnetic moment, g is the gyromagnetic factor, J the total angular momentum and B the magnitude of the applied magnetic field.

**Fig. 6:**
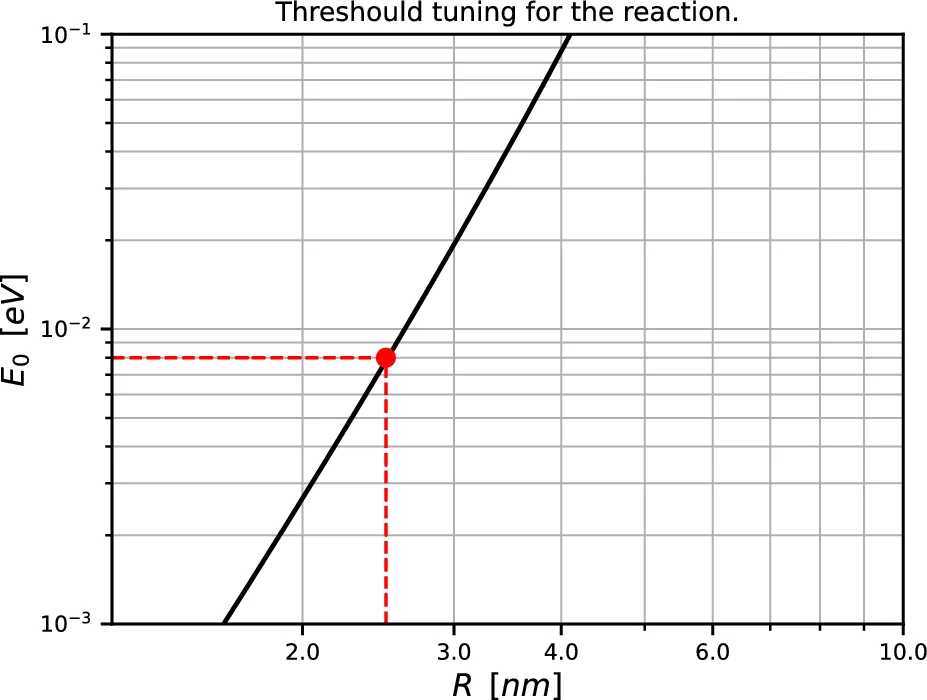
Tuning of the necessary deposited energy to trigger the reaction considering $E_{Zee} = 10^{-3}$eV. As can be seen for a radius of *R ≈ 3*nm, which is around the value for a SMM of Dy$^{\text {III}}$, the energy deposition is close to $10^{-2}$eV as shown by the red dot and lines.

These nanomagnets can go from one state to another via various relaxation mechanisms^35,38,39^. The most common relaxation processes operating in SMMs are the ones that occur through spin-phonon coupling (phonon being the quantum of the vibration mode of the molecules), namely Orbach, Raman and direct processes. Also, we have those that occur due to the quantum nature of the materials, generally quantum tunnelling of the magnetization and thermally assisted relaxation processes^39^. For our case, we are working with lanthanides, f-block metals^35^ in which the important mechanisms are Orbach and Raman^40^. These involve two-phonon-assisted processes, while the others can involve a single or two phonons. For the Orbach relaxation process, the system overcomes the entire barrier *U* (See Fig. 4b) by absorbing phonons from the crystal lattice containing the exact energy $\hbar \omega _i$ to jump from one of the ground sublevels ($E_S = \pm S$) to the highest excited sublevel ($E_S = 0$), with $\omega _i$ being the frequency of the incoming phonons, and so, from this excited state, the system will relax to either the state with $E_S =- S$ or the state with $E_S=+S$ emitting new phonons with energy $\hbar \omega _e$, $\omega _e$ being the frequency of the emitted phonon. Therefore, the energy difference between the absorbed and emitted phonon will correspond to the energy difference between the $\pm E_S$ sublevels in the ground state. The Orbach process does not occur between the highest excited states. Instead, this process usually occurs between the first or second excited states. So, a large phonon energy is required for this process to occur, and it usually operates at the highest temperatures. However, for the Raman, the assisted process is driven by the inelastic dispersion of phonons. In this process, the molecule will absorb a phonon with energy $\hbar \omega _i$, reach a virtual excited state, and emit another phonon with energy $\hbar \omega _e$. In that way, the energy difference between the two phonons will correspond to the energy difference between the $\pm E_S$ sublevels in the ground state.

In fact, all these assisted processes happen due to spin-phonon interactions that require energy in the form of thermally assisted energy. For example, a recent study shows, through ab-initio calculation for a Dy${}^{\text {III}}$ based molecule^40^, that the two relaxation processes that dominate are: Orbach and Raman for high and low temperatures, respectively. As we are interested in the absorption mechanism that injects sufficiently large energies that are bigger than the energy barrier, we consider the Orbach relaxation description (for a review, see^35^),

4 $$\begin{aligned} \tau _R \approx \tau _0\exp \left( \frac{U - \frac{1}{2}\Delta E_{\text {Zee}}}{\Delta T} \right) , \end{aligned}$$

in which *U* is the size of the energy barrier related directly to *D* and $\Delta E_{\text {Zee}}$ is the stored Zeeman energy, $\tau _0$ is the attempt time related to each type of SMM^41^, and *Δ T* is the temperature difference. Furthermore, as pointed out in^42^.

To develop such a macroscopic detector, the long-term stability of the SMM must be evaluated by estimating the baseline spontaneous relaxation time in the absence of external interactions. When the detector is kept at low operating temperatures (*∼ 1* K), thermal relaxation is dominated by the two-phonon Raman process. This process is described by the power law $\tau ^{-1} = D T^n$, where *T* is the temperature, and *n* and *D* are intrinsic properties of the specific SMM complex. Thus, independent of the external magnetic field. To estimate these values, we extracted relaxation data from^2^ and^40^ (as^1^ does not report explicit lifetimes for the molecule in question). In the low-temperature Raman regime, both datasets confirm the standard theoretical expectation exhibiting an exponent of *n = 9*. The extracted coefficients differ by roughly one order of magnitude: $D_{\text {Dinuclear}} \sim \mathcal {O}(10^{-6})\text { s}^{-1}\text {K}^{-9}$ for the complex in^2^, and $D_{\text {Simulation}} \sim \mathcal {O}(10^{-5})\text { s}^{-1}\text {K}^{-9}$ for the simulated system in^40^. Adopting the experimental value of $D \sim 10^{-6}\text { s}^{-1}\text {K}^{-9}$, we find that at an operating temperature of 1K, the metastable lifetime exceeds two days. Furthermore, if the cryostat temperature is lowered to 0.5K, the steep $T^9$ scaling extends the spontaneous lifetime to over a year, ensuring stability over the desired period of time. For this reason, we report our findings for kg-day and kg-year exposure.

Since these molecules might be in a metastable state, as shown in Fig. 4b, if a *O*(1) fraction of SMMs is kept in this state, which can be achieved if the system is maintained at sufficiently low temperatures (*∼ 1* K), we can use them as a means of directly detecting dark matter. The relaxation of these molecules from the metastable state and thus the release of the stored Zeeman energy in the form of thermal energy induces the relaxation of neighboring molecules and so on, as shown in Fig. 1, which sketches the deflagration mechanism known as magnetic avalanche. This process is known and reported and is at the heart of the system’s use as a detector and can be measured using micron-sized Hall sensors^28,41^.

**Fig. 7:**
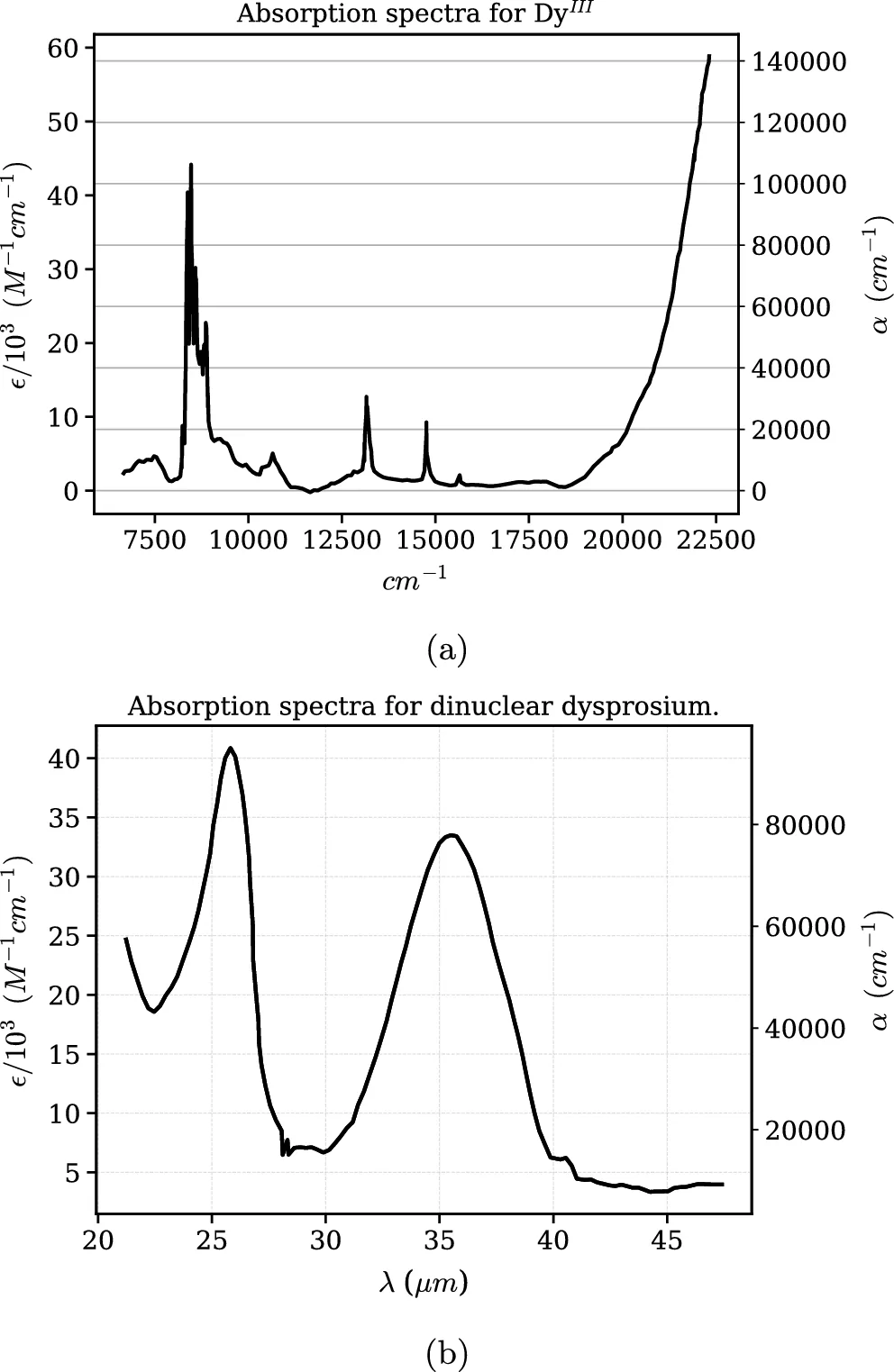
Absorption spectra of both molecules are considered in the analysis. (**a**) the solid-state absorption spectra of UV-vis-NIR for the crystal **1-(P)** as reported from^1^. The x-axis is the wavenumber in $cm^{-1}$ and the y-axis is the absorptivity in $cm^{-1}$. (**b**) the absorption spectra of UV-vis light as reported from^2^. This is the curve that corresponds to complex Dy$_2$(dbm)$_4$(OQ)$_2$(CH$_3$OH)$_2$ synthesized by 8-hydroxyquinoline and dibenzoylmethanate (dbm) ligands. The x-axis corresponds to the wavelength of light, and the left y-axis corresponds to the molecular absorption coefficient and the right y-axis to the absorption coefficient that directly relates to the cross-section via Eq. (10).

**Fig. 8:**
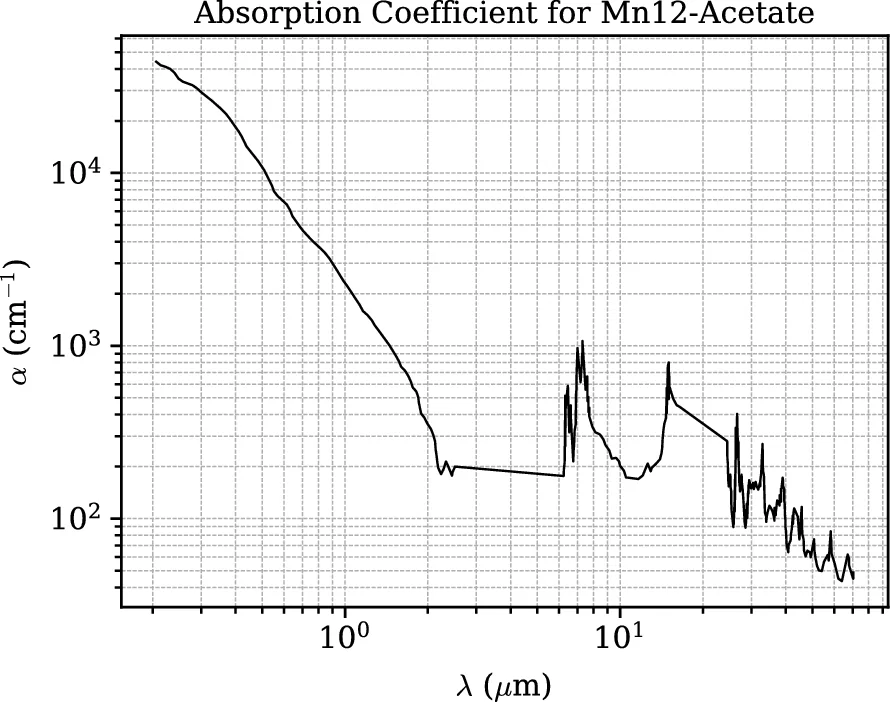
Absorption coefficient for the Mn12-Acetate, which were taken from^3,4^, considered in the calculation of the kinetic mixing of^5^ and on the *axion*-photon coupling of our work. The flat region in the wavelength interval of $10^{0}< \lambda < 10^1$ is an interpolation due to the lack of data.

Magnetic avalanche is a process in which the rapid release of magnetic energy occurs along a given neighbourhood, in a similar way to a cascade. This process usually occurs in solar flares^43^, molecular magnetic materials^41^ and in some types of diodes^44^. For molecular crystals, it is possible to demonstrate this phenomenon through local measurements with temporal resolution of the rapid magnetization reversal in millimeter-sized single crystals of Mn-12 acetate^41^. In this case, the magnetic avalanche takes the form of a thin interface between regions of opposite magnetization. This interface propagates throughout the crystal at a constant speed for a fixed applied field. In fact, the propagation of this interface alters the local magnetization of the molecule within the crystal (see Fig. 1) in a similar way to a cascade. Another way to understand this phenomenon is that it is quite analogous to the propagation of a flame front (deflagration) through a flammable chemical substance.

In order for SMMs to be viable detectors, the efficiency of converting the absorption of a particle, e.g., into a macroscopic signal like an avalanche, must consider at least three factors: intermolecular coupling, crystal size, and disorder. Intermolecular coupling, or predominantly dipolar magnetic coupling in SMMs, such as Mn-12, is the mechanism by which information of a spin reversal is transmitted to neighbors. Depending on the SMM used as a detector, these mechanisms may change, since they depend on the relaxation time. However, the size of the particular crystal must be taken into account in the experimental setup. There is a competition between the heat generated by the spin reversal and the heat dissipated by the external thermal bath in the sample; very small crystals have a high surface-to-volume ratio, meaning that heat flows from the crystal faster than it can force the neighbouring molecules to reverse their spins. Therefore, below a certain critical size, the crystal cannot sustain the thermal avalanche. Thus, larger crystals retain heat through lattice vibrations, phonons, ensuring propagation. The typical size for a Mn-12 acetate is of 2.5 × 0.5 × 0.5 mm^45^. On the other hand, no SMM crystal is perfect, as there may be dislocations in the lattice, molecules that have lost solvent ligands, or misalignment of the magnetic anisotropy axis. Disorder creates sites in the crystal where the energy barrier, *U*, for spin reversal is locally lower than that of other sites in the lattice. These anomalous molecules act as nucleation points, namely, a particle that reaches the vicinity of a defect needs to deposit much less energy to initiate the avalanche, drastically altering the threshold at different points in the crystal. Therefore, the procedure to synthesize the SMM is an important point from the experimental side, as one would need a crystal with fewer defects to control the energy threshold.

Specifically, the absorption of dark photons or axions will excite a molecular spin state, followed by magnetic relaxation across the anisotropy barrier via Orbach or Raan relaxation for lanthanides, or direct processes as in Mn-12. One way to detect such avalanches is via graphene hall sensors placed near the samples as done in^45^, where they detected such phenomena due to a single quantum of energy in a Mn-12 sample. The detection of a spin avalanche is seen as a sudden change in the measured magnetic field of the sample in time; another possibility also pointed out in^45^, is the measurement of the avalanche with a thermometer positioned near a sample of SMM. If an avalanche happens, a sudden increase in temperature happens at the exact same time as a change in the magnetic field of the sample. Although different molecules, the dominant relaxation mechanism for the Mn-12 and Dysprosium samples is the Orbach mechanism, given by the Arrhenius law given in Eq. (4); therefore, we expect that the results found in^45^ could be extended to the Dysprosium-based SMM. That speaks about the feasibility of our proposal.

Another possibility is also pointed out in^46^, where they also use a Mn-12 sample applying SQUID Magnetometry, X-band Electron Paramagnetic Resonance (EPR), Muon Spin Rotation ($\mu _{\text {SR}}$) and Nuclear Magnetic Resonance, for a precise quantum measure of the signal. Nevertheless, the exact setup and the experimental readout for using lanthanides, such as dysprosium based SMM, for detecting a single quantum of energy or dark matter are to be determined.

The velocity of magnetic avalanches can be given by a crude estimate using the heat equation^41^ for a sample of Mn12-acetate, and can be used to assess the sensitivity of the reaction to that of the deposited energy. The velocity can be determined using Hall sensors that measure the magnetic field in a direction perpendicular to the applied field. Our proposal relies on a different sample, but the same relaxation process drives the deflagration mechanism once DM hits the detector, that is the Orbach routine^40,41^. Hence, we can use this velocity to assess the deposited energy of the DM particle,

5 $$\begin{aligned} v \sim \sqrt{\frac{\kappa }{\tau _0}}\exp \left( - \frac{U - \frac{1}{2}\Delta E_{\text {Zee}}}{\Delta T} \right) , \end{aligned}$$

where *κ * is the thermal diffusivity of the SMMs. This magnetic avalanche propagates as the narrow interface through the crystal at a constant velocity^28,41^. The temperature difference can be put in terms of the deposited energy $E_0$ from the DM particle (see Sec. 3), which can serve as a probe for the signal; this feature can be seen in Fig.5.

## Setting up the detector

In order to find the necessary energy to trigger the magnetic avalanche reaction in a SMM detector, we can follow^5^ in comparing the characteristic time of relaxation, $\tau _R$ for an Arrhenius law ($\tau _R \approx \tau _0 \exp {(\frac{U - \frac{1}{2}\Delta E_{\text {Zee}}}{\Delta T})}$, where $\tau _0$ is a constant related to each SMM and it may vary from $10^{-12}-10^{-9}$, $\Delta E_{\text {Zee}} = 2 \mu _B g_J S B$ is the Zeeman splitting, *U* the size of the energy barrier and *Δ T* the difference in temperature) with the time heat takes to dissipate in the detector, $\tau _D$.

The justification for choosing the Arrhenius law is based on two assumptions: first that these molecules have a stored Zeeman energy of $E_{Zee} = 10^{-3}$eV, and that upon relaxing all Zeeman energy heats the avalanche front, corresponding to a temperature greater than 10K. In^40^ the molecule considered has a cross over from Raman, which is described by a power law $C T^n$ to Orbach, which is described by the Arrhenius law, at 10 K. Since their considerations take into account an SMM with energy barrier greater than ours, it is reasonable to assume that the propagation of the avalanche is completely dictated by the Orbach process.

Considering that we need the SMM to flip before heat dissipates, we need $\tau _R < \tau _D$. With this imposition, we can sense the minimum energy needed if we assume that the SMM will have a Debye behavior for its temperature dependence, which is the observed behavior for SMMs at low temperatures^47^. This approximation yields the following,

6 $$\begin{aligned} E_0 \gtrsim \frac{c_0 R^3(U- \frac{1}{2}\Delta E_{\text {Zee}})^4}{\ln {\left( \frac{R^2}{\tau _0 \kappa }\right) }}, \end{aligned}$$

where $c_0$ and *R* are the SMM’s volume-specific heat capacity and radius.

**Fig. 9:**
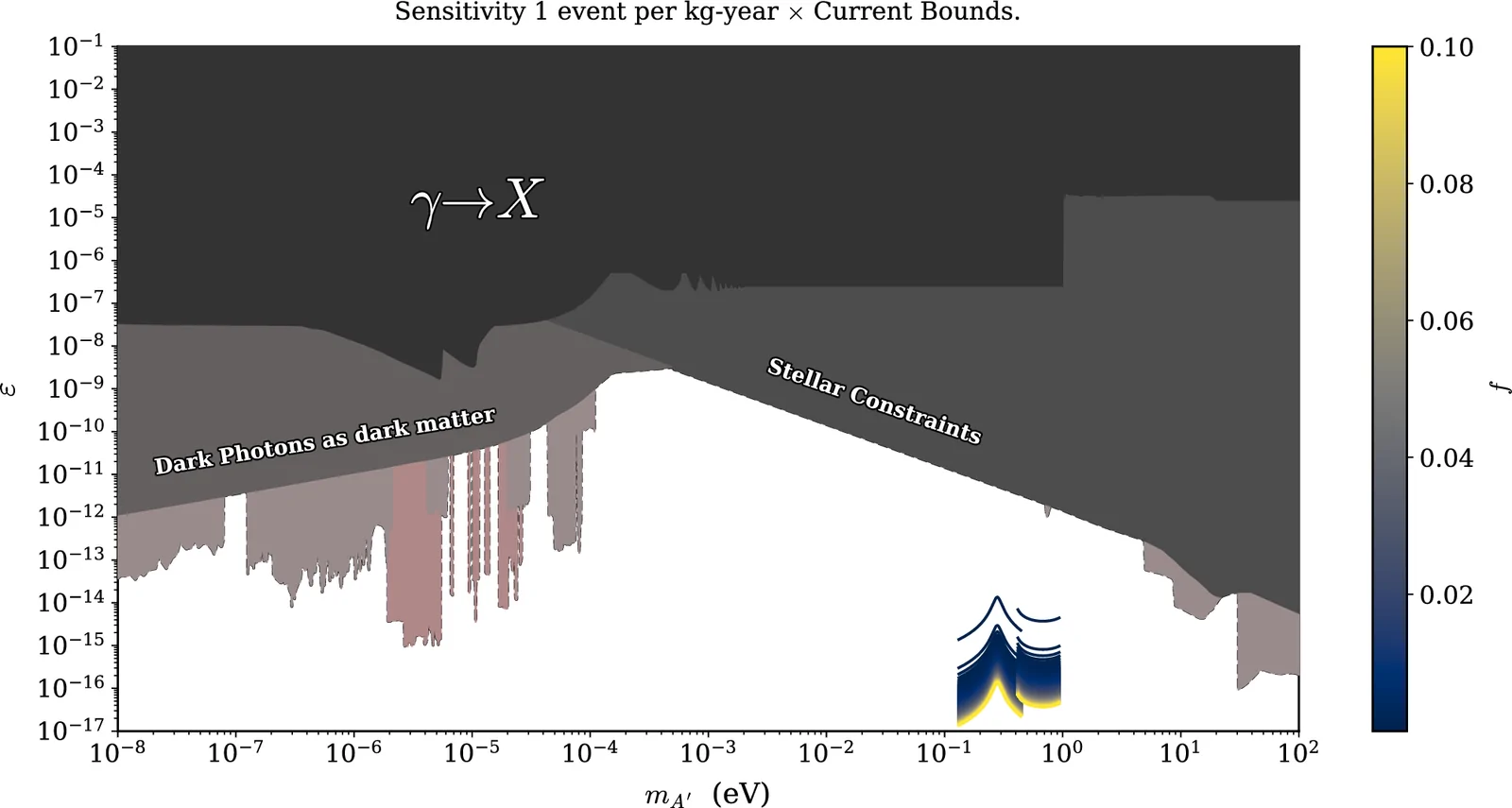
Expected sensitivity to the kinetic mixing, *\varepsilon *, which governs the interaction with SM fermions. We varied $f=10^{-5}-0.1$. Clearly, dysprosium can be a promising laboratory for *dark photons* with the potential to cover a few orders of unexplored parameter space.

Eq. (6) places a lower bound on the energy threshold as a function of *R* for SMMs. Using the data reported in^2^ for the dinuclear Dysprosium illustrated in Fig. 2 that features $\tau _0 \sim 4 \times 10^{-9}$s, *U = 109.5*K and $\kappa \sim 10^{-7} \text {m}^2/\text {s}$ at 1*K*^2^, while for Dy$^{\text {III}}$^1^ does not report the value of $\tau _0$, we adopted the same value.

The results are shown in Fig.6 considering a Zeeman energy of $E_{Zee} = 10^{-3}$eV, which corresponds to using $g_Z \sim 20$^40^, *B = 1T*, *S = 15/2*. There we can see that for an SMM molecule of radius *∼ (2-3)*nm, the threshold energy to trigger the reaction will be $\sim 10^{-2}$eV. Given that the DM velocity in our galaxy is of order $v\sim 10^{-3}$, the average deposited energy is approximately $\sim 10^{-7} - 10^{-6}$eV, which is insufficient to trigger the reaction. To account for this, we consider that only a fraction *f* of DM particles will have sufficient energy to initiate the reaction. These particles may originate from a class of physical processes that impart additional energy to DM, a scenario known as Boosted Dark Matter (BDM)^20–23,48,49^.

## Results

In this section, we demonstrate the new physics reach of this proposed detection method for the *dark photon* and *axion* models. In particular, we will assess the sensitivity to the kinetic mixing parameter *\varepsilon * for the *dark photon* dark matter (DPDM), $A_\mu '$, and the *axion*-photon coupling, $g_{a\gamma \gamma }$, for *axion* model. Starting with the *dark photon* model, it is well-known that the kinetic mixing term between the photon and the dark photon in Eq. (1) induces an interaction between with SM fermions described as follows,

7 $$\begin{aligned} \mathcal {L}_{\text {DP}} \supset e\varepsilon A'_\mu J^\mu , \end{aligned}$$

which is very similar to QED, but with the electric charge suppressed by the kinetic mixing angle *\varepsilon *. Consequently, the interaction cross-section of SM fermions and the dark sector gauge boson can be expressed as the QED cross-section rescaled by $\varepsilon ^2$^29^,

8 $$\begin{aligned} \sigma _{A'} = \varepsilon ^2 \sigma ^\gamma . \end{aligned}$$

We can reinterpret the average absorption cross section for the *dark photon* model^5,50^,

9 $$\begin{aligned} \langle n \sigma _{\text {abs}}(m_{A'})v\rangle \approx \varepsilon ^2 \langle n \sigma ^\gamma _{\text {abs}} (c = 1)\rangle . \end{aligned}$$

This approximation arises because the mixing angle for absorption is rescaled by the relative permittivity of the SMM, which can be approximated as unity^5^. This approximation, alongside the rescaling of the *dark photon* cross-section, is helpful because the actual cross-section for photons being absorbed in materials is known^5^,

10 $$\begin{aligned} \sigma ^\gamma _{\text {abs}} = \frac{\alpha }{n}, \end{aligned}$$

where *α * is the absorption coefficient of the material and *n* its number density. While for the *axion*, its coupling to the photon is via a dimension five operator in Eq.(2), which leads to^34^,

11 $$\begin{aligned} \mathcal {L}_{\text {a}} = g_{a \gamma \gamma }\, a \,\vec {E} \cdot \vec {B}, \end{aligned}$$

where $g_{a\gamma \gamma }$ has dimension of Energy${}^{-1}$. We can determine the total rate of absorption of an *axion* in a magnetized medium per unit exposure,

12 $$\begin{aligned} R \simeq \left( \frac{g_{a \gamma \gamma } B_0}{m_a} \right) \frac{\rho _{\text {DM}}}{\rho _\text {T}} \text {Im} \left[ -\frac{1}{\kappa (m_a)} \right] , \end{aligned}$$

where $m_a$ is the *axion*’s mass, $\rho _\text {DM}$ and $\rho _\text {T}$ are the DM local density, and the target’s density, $B_0$ is the applied magnetic field in the medium, and $\kappa (m_a)$ is the dielectric function evaluated at the *axion*’s mass^33,51–53^ and,

13 $$\begin{aligned} g_{a \gamma \gamma } \sim \frac{\varepsilon m_a}{B_0}. \end{aligned}$$

Via Eq. (13), we translate the expected sensitivity to the *axion*-photon coupling. Eq. (13) also shows that the *axion*-photon coupling is amenable to the strength of the applied magnetic field, contrary to the kinetic mixing sensitivity, which has no dependence at all in our case. This is a considerable feature for the *axion*, because we can improve the sensitivity through strong magnetic fields, while reducing the energy threshold in Eq. (6). Thus, if we lower the threshold enough, we might not need to work in the framework of Boosted Dark Matter anymore. Nonetheless, to be consistent with the *dark photon* case, we will consider both with the same value of applied magnetic field: *B = 1*T. Therefore, there is still a need for the BDM framework as Eq. (6) is model independent.

We need to derive the absorption coefficient, *α *, for the molecules in question to obtain the absorption rate. To do so, we need to use absorbance or transmittance via Beer-Lambert’s law,

14 $$\begin{aligned} A = -\log _{10} e^{-\alpha hl}, \end{aligned}$$

where *h* is the concentration and *l* the thickness of the material under analysis. To obtain data for the molecules considered, we used^1,2^. However, the reported data is in terms of the molecular absorption coefficient as can be seen in the left y-axis of Fig. 7, which relates to Beer-Lambert’s law in a different form,

15 $$\begin{aligned} A = \epsilon \times c \times l, \end{aligned}$$

where *ε * is the molecular absorption coefficient, *c* is the concentration given in mol*/L = M*, which makes $[\epsilon ] =\text {cm}^{-1} \text {M}^{-1}.$ The conversion from one to the other is made considering that the molar concentration can be related to the concentration in Eq. (14) by $c = \frac{\rho \times h}{m}\times \frac{10^3}{N_A}$, for *ρ , m*, and $N_A$ are the density, mass and Avogadro’s number of the molecule. Using the molar mass of the substance in consideration, $\mathcal {M} = \frac{m}{N_A}$, we get the exact relationship between both coefficients,

16 $$\begin{aligned} \alpha = 10^3\ln {10} \frac{\rho }{\mathcal {M}}\epsilon . \end{aligned}$$

Using this equation and the data reported in^1,2^ for the density and molecular mass of the substances, we get the exact values for *α * as a function of wavenumber. The values are shown in the y-axis of Fig. 7, where the shape for *ε * and *α * are the same, because the conversion is independent of the wavelength under consideration. With the exact values for *α * we can then directly connect the reported data with the prospects for the sensitivity of a DPDM particle being absorbed in the detector.

**Fig. 10:**
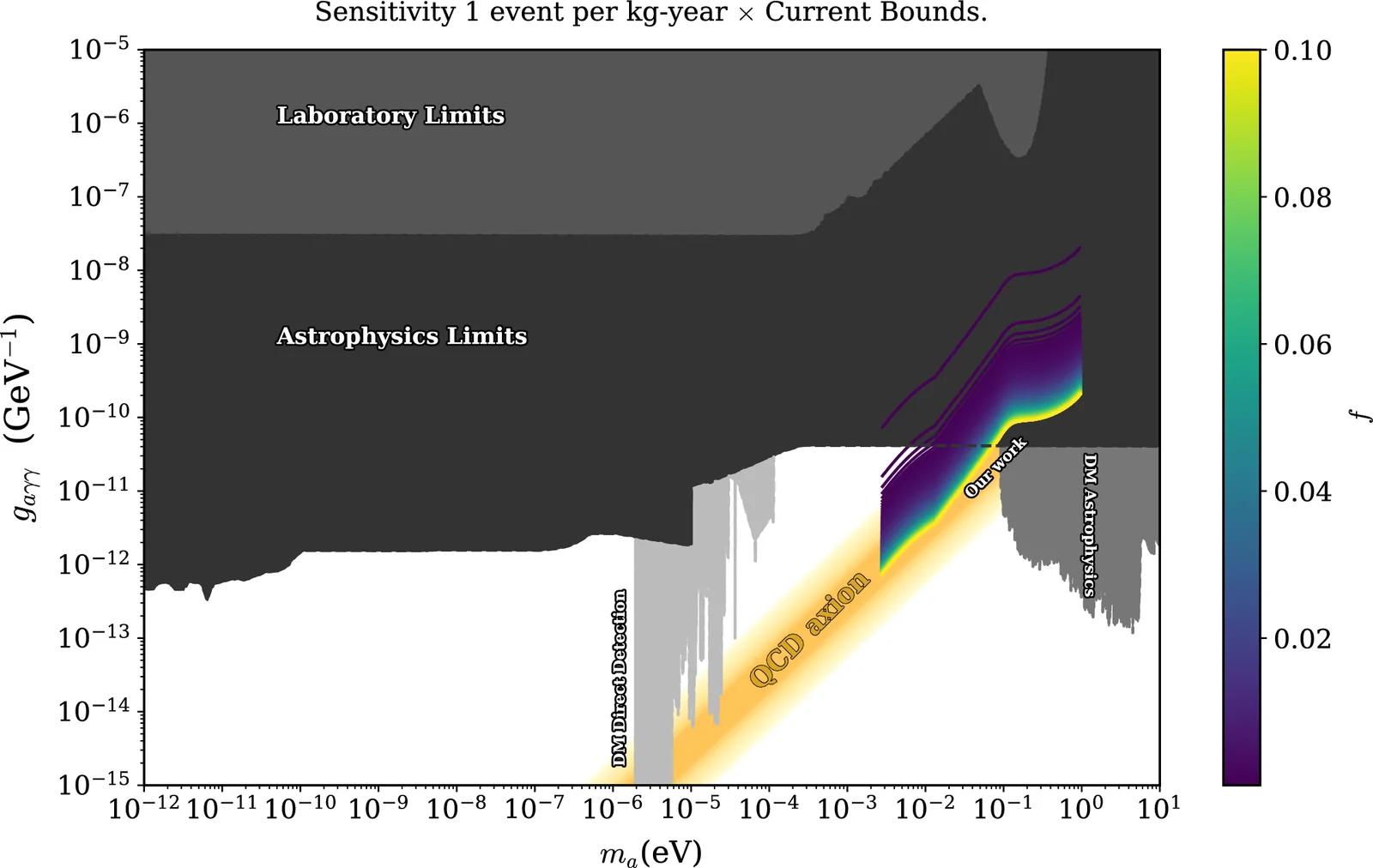
Expected reach to the *axion*-photon coupling overlaid with existing bounds in gray. For $m_a < 10^{-1}$ eV our proposal yields a promising sensitivity. We have varied the fraction of boosted dark matter in the range $f=10^{-5}-0.1$.

**Fig. 11:**
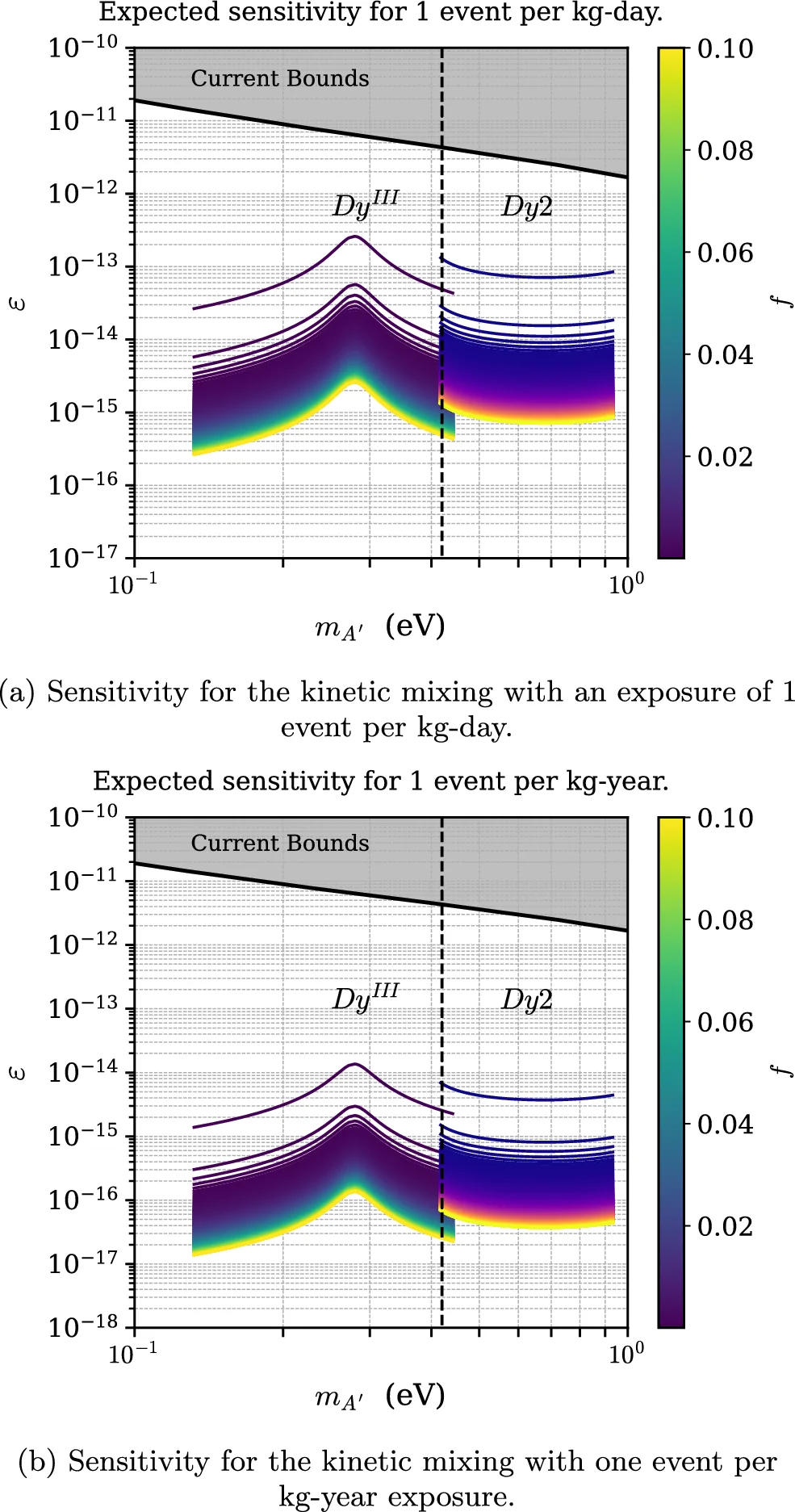
Expected sensitivities for different exposure times for the *dark photon* model assuming: (**a**) 1 event per kg-day; (**b**) one event per kg-year.

The reach is found using the rate of interaction of a DM particle in a detector^6^,

17 $$\begin{aligned} R = \frac{\rho _{DM}}{m_{DM}}\frac{1}{\rho }\langle n \sigma v \rangle , \end{aligned}$$

where $\rho _{DM}$ and $m_{DM}$ are the density and the mass of DM respectively, *ρ * and *n* are the density and number density of the crystal and *v* is the DM velocity, which can be regarded as $v \approx 10^{-3}$, however when we substitute Eq. (9) in Eq. (17) there will be no velocity dependence, therefore when working in the framework of BDM to account for the triggering the reaction as explained in Sec. (), we only need to add on Eq. (17) a new term *f* that account for the fraction of DM in our Solar System that is boosted and reaches the detector, Eq. (17) becomes,

18 $$\begin{aligned} R \simeq f \frac{\rho _{DM}}{m_{DM}}\frac{1}{\rho }\langle n \sigma _{\text {abs}}^\gamma \rangle , \end{aligned}$$

where *f* represents the fraction of DM that will be boosted; this can be seen as a rescaling of $\rho _{DM} \rightarrow f \rho _{DM}$, which essentially states that a fraction of DM in the vicinity of our Solar System will be boosted by some mechanism^20–23,48,49^. We derive the rate by inserting Eq. (9) in Eq. (18) using absorption data plotted in Fig. 7a and b. The expected sensitivity is then calculated for $\omega = m_{A'}$ for a given DM mass in Fig. 11 for $f=10^{-5} - 10^{-1}$ (Fig. 8).

The expected sensitivity in Fig. 9 declines as the amount of BDM available for absorption in the detector decreases . The cusp in the mass region between $10^{-1} - 10^0$ is related to the data in Fig. 7a. As can be seen, the absorption gets close enough to zero at 12000 cm^−1^, and given the kinetic mixing dependence on the absorption coefficient scales as $\propto 1/\alpha ^{\frac{1}{2}}$, it is to be expected to behave as presented in the Fig. 9. Another prompt change can be in scaling the detector mass, given that the rate of observed events in Eq. (18) depends on the exposure, if we increase the detector’s mass we gain a greater sensitivity in the kinetic mixing *\varepsilon *; this scaled is explicitly pointed out in the dependence of the following by setting the exposure to be 1 kg-year or 1 kg-day. Figure 9 explicitly shows the expected sensitivity for an exposure of 1 event per kg-year. We put our findings into perspective with current bounds on *dark photons*^54,55^. In the mass region of interest, the limits neither come directly from direct detection experiments nor from *dark photon* dark matter searches. Nevertheless, the scientific reach of our proposal surpasses that of existing experiments, being able to cover a large region of untouched parameter space.

In particular, the shaded regions are labeled accordingly to the type of experiments in question; $\gamma \rightarrow X$ are laboratory limits in *\varepsilon * coming from light shining through a wall^56–58^, Cavendish-Coulomb^59^; *dark photon* as Dark Matter are the limits for the DP corresponding to the totality of DM^60–63^, and Stellar Constraints come from astrophysics limits^64–66^; the other colored regions without labels refer to Direct Detection experiments^8–10,67^ in the mass region of $10^0 - 10^2$, and other laboratory experiments, such as the measurement of Dark-E fields^68^ or radio frequency cavity searches^69^ for the lower mass region: $10^{-8} - 10^{-4}$. The blue-to-yellow region corresponds to our calculations for the two new proposed molecules: Dy2^1^ and Dy${}^{\text {III}}$^2^.

As aforementioned, we can reinterpret these results for *axions*. Using the absorption spectra for dinuclear dysprosium and assuming 1 event per kg-year of exposure, we ended up probing a region of parameter space already excluded by *axion* searches (*see Appendix*). That said, we adopted the Mn12-acetate, a complex compound of manganese, and repeated the entire procedure described above and derived the expected sensitivity to *axions* as displayed in Fig. 10 adopting a magnetic field $B_0 = 1$ T. The shape of the curve is very similar to that of the *dark photon* model. The break exhibited in the curve of Fig. 10 has to do with the linear dependence in the mass that appears for the coupling, which is not present in the case of the *dark photon* model. The projected sensitivity can be improved using a stronger magnetic field.

We have superimposed the existing limits in the literature^55^. These bounds stem from laboratory searches^70–73^, astrophysics searches for *axions* not as DM^74–85^, astrophysics bounds for *axions* as DM^86–93^, and DM direct detection bounds^94–96^.

The purple-to-yellow colored region is a result of our estimate based on Eq. (13). The expected reach becomes promising for masses $m_a < 10^{-1}$ eV. We emphasize that we can increase the applied magnetic field to improve the sensitivity. From Fig. 10, it is clear that our proposal offers the possibility to probe a sizable region of the QCD *axion* parameter space, with leading sensitivity for *axion* masses between $2\times 10^{-3}$ and $8\times 10^{-2}$ eV. It is worth noticing that we can push down these projections if we either increase the applied magnetic field or consider a higher fraction of BDM than the one considered in the plots.

In summary, by integrating concepts from particle physics, condensed-matter physics, and chemistry, we demonstrate that interdisciplinary approaches can enable genuinely new detection strategies and open access to previously unexplored regions of parameter space.

## Conclusion

The community has been dedicated to testing the compelling WIMP paradigm via accelerators, direct and indirect detection experiments, but null results have been reported thus far. This has motivated the community to explore other dark matter candidates and novel detection mechanisms. In this spirit, we propose to use Single Molecule Magnets as dark matter laboratories, particularly focused on the *dark photon* and *axion* models.

We highlight that we carried out a theoretical sensitivity study based on negligible background sources, a nearly noise-free experiment given the measurement conducted in^45^; in^45^ they use Mn-12 for alpha particle detection, and therefore to further assess the feasibility and the actual noise for Dysprosium based SMMs one would need to prepare the same setup with it, nevertheless we quote it here to show that such almost noise free experiment using a magnetic molecule is possible. The presence of any radiative background with energy greater than $10^{-2}$ eV could in principle trigger an avalanche process and alter our quantitative results.

Single Molecule Magnets function as metastable systems for relatively long periods. Dark-matter absorption triggers accelerated relaxation from these states, producing detectable magnetic avalanches. Using dysprosium- and manganese-based molecules, we computed reaction rates for dark-photon and axion interactions, deriving projected sensitivities. Our analysis demonstrates that dysprosium SMMs improve sensitivity to dark photons by orders of magnitude, while manganese systems probe previously inaccessible regions of the QCD axion parameter space.

Our work builds a bridge between chemistry, condensed-matter physics, and particle physics for dark-matter detection in studying the theoretical sensitivity of these magnets to detecting dark photons, and axions. Nevertheless, we understand that there are challenges in the realization of this detector such as the scalable synthesis of high quality crystals and the engineering of the experimental apparatus remain open challenges for future R &D. 

## Figures and Tables

**Fig. 12:**
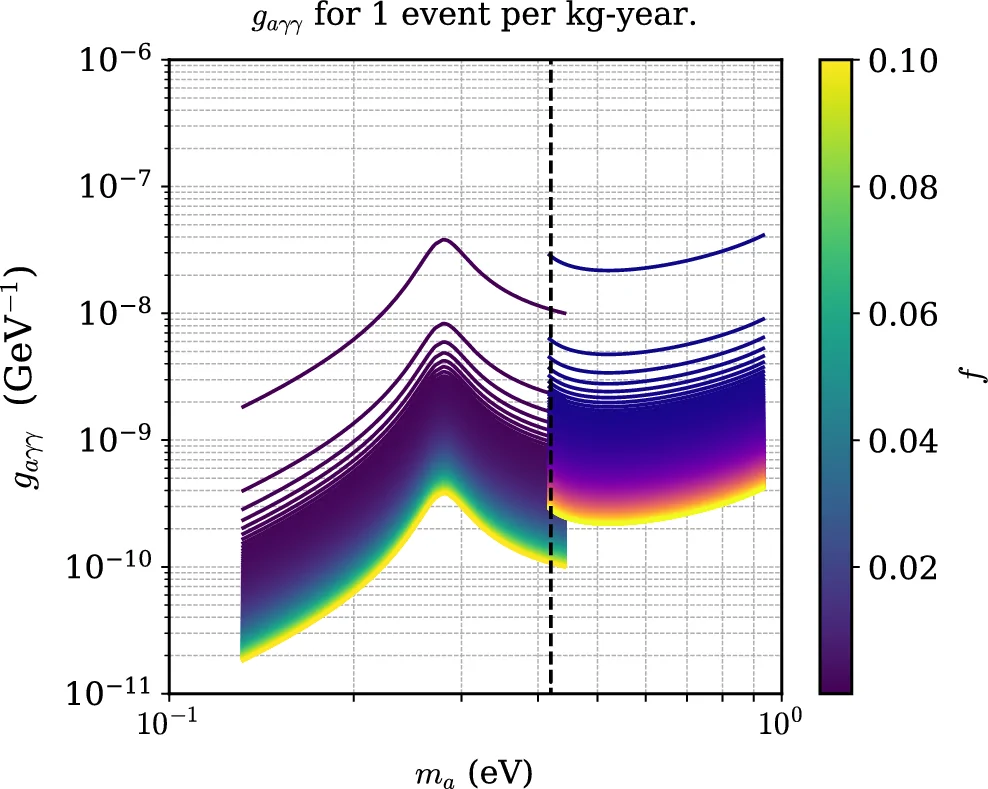
Expected reach to *axion*-photon coupling for the dysprosium molecules assuming 1 event per kg-year.

**Fig. 13:**
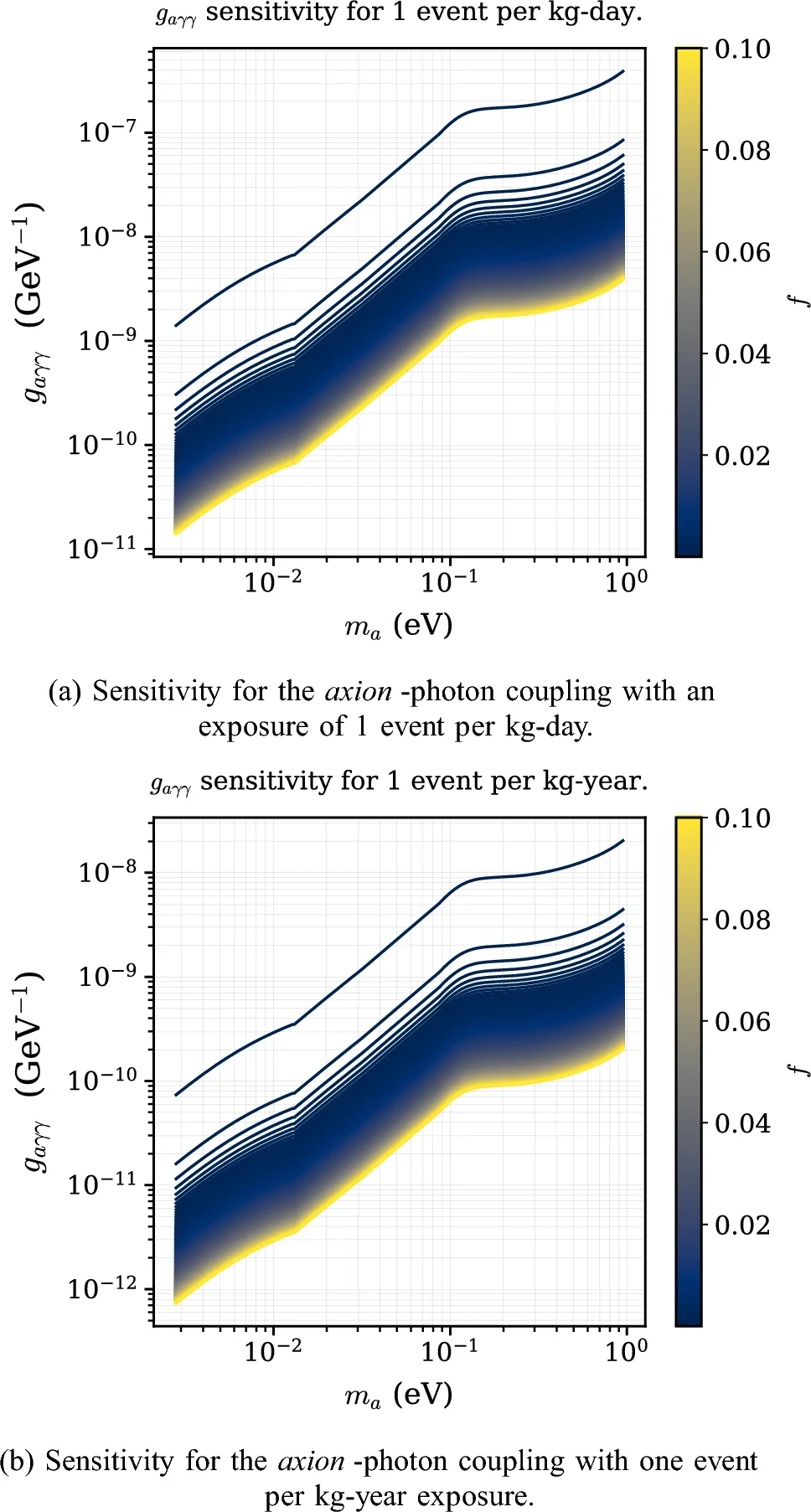
Projected reach of a manganese compound to the *axion*-photon coupling using 1 one event per kg-day (**a**), and 1 one event per kg-year (**b**).

## Data Availability

All data were taken from [1, 2] for the Dark-Photon calculation and from [3–5] for the axion absorption. All the calculation and code are available at the GitHub link https://github.com/robalvess/SMM-DM.

## Appendix A

In this section, we present the sensitivity with an exposure of 1 event per kg-day to both *dark photon* and *axion* dark matter particles. These plots are an extension of the results are exhibited in Figs. 9, 10 but offer a clearer vision of the scientific reach in comparison with existing bounds and the role of each compound.

In Fig. 11a–b we display the sensitivity for two different exposures using dysprosium molecules. Notoriously, with a larger exposure, a greater reach is obtained. This behavior is expected since the kinetic mixing scales as $(\text {kg-time})^{-1/2}$. These findings were derived using Eq. (18) for $f=10^{-5}- 0.1$. From Fig. 11a–b, we solidly show that such molecules offer a powerful platform for probing physics beyond the Standard Model.

Using the same molecules, we also derived the sensitivity to the *axion*-photon coupling. The results are summarized in Fig. 12 for an exposure of 1 event per kg-year. The parameter space covered in Fig. 12 has already been excluded. Thus, we decided to use a different molecule, Mn12-acetate, which is a complex compound of manganese, and repeat the same procedure. The expected reach is shown in Fig. 13a–b for different exposures, similar to the previous figures. Our finding is based on Eq. (13) and the sensitivity projected for kinetic mixing using Mn12-acetate^5^. The shape of the curve is very similar to that of the *dark photon* model. The small tilt in Fig. 13a–b is due to the linear dependence in the mass that appears for the coupling, which is not present in the case of the kinetic mixing. We have adopted a magnetic field $B_0 = 1$T, but a better sensitivity can be achieved with larger magnetic fields.

Our findings were based on two compounds, and for this reason, there is a visible break in the curves. In particular, we observe that dy2 covers larger masses compared to the $Dy^{III}$.

## References

[1] Raju, M. S. et al. Magneto-chiral dichroism in a one-dimensional assembly of helical dysprosium(iii) single-molecule magnets. *Inorganic Chemistry* **62**, 17583–17587. 10.1021/acs.inorgchem.3c03204 (2023). 37856861

[2] Shen, H.-Y., Wang, W.-M., Gao, H.-L. & Cui, J.-Z. Near-infrared luminescence and smm behaviors of a family of dinuclear lanthanide 8-quinolinolate complexes. *RSC Advances* **6**, 34165–34174. 10.1039/C6RA02656G (2016).

[3] Sushkov, A. B. et al. Magnetic field effects on the far-infrared absorption in -acetate. *Phys. Rev. B* **63**, 214408. 10.1103/PhysRevB.63.214408 (2001).

[4] Oppenheimer, S. M., Sushkov, A. B., Musfeldt, J. L., Achey, R. M. & Dalal, N. S. Diffuse optical excitations in -acetate. *Phys. Rev. B* **65**, 054419. 10.1103/PhysRevB.65.054419 (2002).

[5] Bunting, P. C., Gratta, G., Melia, T. & Rajendran, S. Magnetic Bubble Chambers and Sub-GeV Dark Matter Direct Detection. *Phys. Rev. D* **95**, 095001 (2017) arXiv:1701.06566.

[6] Cirelli, M., Strumia, A. & Zupan, J. Dark Matter (2024). arXiv:2406.01705.

[7] Green, A. M. Primordial black holes as a dark matter candidate - a brief overview. *Nucl. Phys. B* **1003**, 116494 (2024) arXiv:2402.15211.

[8] Schumann, M. Direct Detection of WIMP Dark Matter: Concepts and Status. *J. Phys. G* **46**, 103003 (2019) arXiv:1903.03026.

[9] Aprile, E. et al. First Dark Matter Search with Nuclear Recoils from the XENONnT Experiment. *Phys. Rev. Lett.* **131**, 041003 (2023) arXiv:2303.14729. 37566859 10.1103/PhysRevLett.131.041003

[10] Aprile, E. Search for Light Dark Matter in Low-Energy Ionization Signals from XENONnT. *Phys. Rev. Lett* **134**, 161004 (2025) arXiv:2411.15289. 40344124 10.1103/PhysRevLett.134.161004

[11] Gaskins, J. M. A review of indirect searches for particle dark matter. *Contemp. Phys* **57**, 496–525 (2016) arXiv:1604.00014.

[12] Albert, A. Searching for Dark Matter Annihilation in Recently Discovered Milky Way Satellites with Fermi-LAT. *Astrophys. J* **834**, 110 (2017) arXiv:1611.03184.

[13] Boveia, A. & Doglioni, C. Dark Matter Searches at Colliders. *Ann. Rev. Nucl. Part. Sci.* **68**, 429–459 (2018) arXiv:1810.12238.

[14] Perez Adan, D. Dark Matter searches at CMS and ATLAS. In 56th Rencontres de Moriond on Electroweak Interactions and Unified Theories (2023) arXiv:2301.10141.

[15] Wang, J.-W. Blazar-boosted dark matter: Novel signatures via elastic and inelastic scattering. *Phys. Rev. D* **112**, 055004 (2025) arXiv:2503.22105.

[16] Essig, R., Manalaysay, A., Mardon, J., Sorensen, P. & Volansky, T. First Direct Detection Limits on sub-GeV Dark Matter from XENON10. *Phys. Rev. Lett.* **109**, 021301 (2012) arXiv:1206.2644. 23030151 10.1103/PhysRevLett.109.021301

[17] Essig, R., Volansky, T. & Yu, T.-T. New Constraints and Prospects for sub-GeV Dark Matter Scattering off Electrons in Xenon. *Phys. Rev. D* **96**, 043017 (2017) arXiv:1703.00910.

[18] Ghosh, D. K., Gupta, T., Heikinheimo, M., Huitu, K. & Jeesun, S. Boosted dark matter driven by cosmic rays and diffuse supernova neutrinos. *Phys. Rev. D* **111**, 063019 (2025) arXiv:2411.11973.

[19] Agashe, K., Cui, Y., Necib, L. & Thaler, J. (In)direct Detection of Boosted Dark Matter. *JCAP* **10**, 062 (2014) arXiv:1405.7370.

[20] Bringmann, T. & Pospelov, M. Novel direct detection constraints on light dark matter. *Phys. Rev. Lett.* **122**, 171801 (2019) arXiv:1810.10543. 31107056 10.1103/PhysRevLett.122.171801

[21] Das, A., Herbermann, T., Sen, M. & Takhistov, V. Energy-dependent boosted DM from DSNB. *PoS* **NOW2024**, 014 (2025).

[22] Herbermann, T., Lindner, M. & Sen, M. Attenuation of cosmic ray electron boosted dark matter. *Phys. Rev. D* **110**, 123023 (2024) arXiv:2408.02721.

[23] Das, A., Herbermann, T., Sen, M. & Takhistov, V. Energy-dependent boosted dark matter from diffuse supernova neutrino background. *JCAP* **07**, 045 (2024) arXiv:2403.15367.

[24] Hochberg, Y., Lin, T. & Zurek, K. M. Absorption of light dark matter in semiconductors. *Phys. Rev. D* **95**, 023013 (2017) arXiv:1608.01994.

[25] Kahn, Y. & Lin, T. Searches for light dark matter using condensed matter systems. *Rept. Prog. Phys.* **85**, 066901 (2022) arXiv:2108.03239. 10.1088/1361-6633/ac5f63 35313296

[26] Herrera, F. & Spano, F. C. Dark Vibronic Polaritons and the Spectroscopy of Organic Microcavities. *Phys. Rev. Lett.* **118**, 223601 (2017) arXiv:1610.04252. 28621976 10.1103/PhysRevLett.118.223601

[27] Hochberg, Y., Pyle, M., Zhao, Y. & Zurek, K. M. Detecting Superlight Dark Matter with Fermi-Degenerate Materials. *JHEP* **08**, 057 (2016) arXiv:1512.04533.

[28] Friedman, J. R. & Sarachik, M. P. Single-molecule nanomagnets. *Annual Review of Condensed Matter Physics* **1**, 109–128. 10.1146/annurev-conmatphys-070909-104053 (2010).

[29] Fabbrichesi, M., Gabrielli, E. & Lanfranchi, G. *The Physics of the Dark Photon: A Primer* (Springer International Publishing, 2021). 10.1007/978-3-030-62519-1.

[30] Peccei, R. D. & Quinn, H. R. CP Conservation in the Presence of Instantons. *Phys. Rev. Lett.* **38**, 1440–1443 (1977).

[31] Preskill, J., Wise, M. B. & Wilczek, F. Cosmology of the Invisible Axion. *Phys. Lett. B* **120**, 127–132 (1983).

[32] Kim, J. E. & Carosi, G. Axions and the Strong CP Problem. *Rev. Mod. Phys.* **82**, 557–602 (2010). [Erratum: Rev.Mod.Phys. 91, 049902 (2019)], arXiv:0807.3125.

[33] Berlin, A. & Trickle, T. Absorption of Axion Dark Matter in a Magnetized Medium. *Phys. Rev. Lett.* **132**, 181801 (2024) arXiv:2305.05681. 38759193 10.1103/PhysRevLett.132.181801

[34] Navas, S. et al. Review of particle physics. *Phys. Rev. D* **110**, 030001 (2024).

[35] Zabala-Lekuona, A., Seco, J. M. & Colacio, E. Single-molecule magnets: From mn12-ac to dysprosium metallocenes, a travel in time. *Coordination Chemistry Reviews* **441**, 213984 (2021). URL https://www.sciencedirect.com/science/article/pii/S0010854521002587.

[36] Orts-Arroyo, M., Rojas, C., Moliner, N. & Martínez-Lillo, J. Lipoic acid-functionalized hexanuclear manganese(iii) nanomagnets suitable for surface grafting. *International Journal of Molecular Sciences* **24**, 8645 (2023). 37239996 10.3390/ijms24108645 PMC10218575

[37] Sessoli, R., Gatteschi, D., Caneschi, A. & Novak, M. Magnetic bistability in a metal-ion cluster. *Nature* **365**, 141–143 (1993).

[38] Moreno-Pineda, E., Taran, G., Wernsdorfer, W. & Ruben, M. Quantum tunnelling of the magnetisation in single-molecule magnet isotopologue dimers. *Chem. Sci.* **10**, 5138–5145. 10.1039/C9SC01062A (2019). 31183066 PMC6524608

[39] Abragam, A. & Bleaney, B. *Electron Paramagnetic Resonance of Transition Ions*. Oxford Classic Texts in the Physical Sciences (Oxford, 2012). URL https://books.google.com.br/books?id=ASNoAgAAQBAJ.

[40] Briganti, M. et al. A complete ab initio view of orbach and raman spin–lattice relaxation in a dysprosium coordination compound. *J. Am. Chem. Soc.* **143**, 13633–13645 (2021). 10.1021/jacs.1c05068. PMC8414553 34465096

[41] Suzuki, Y. et al. Propagation of avalanches in -acetate: Magnetic deflagration. *Phys. Rev. Lett.* **95**, 147201 (2005) (**10.1103/PhysRevLett.95.147201**). 16241690 10.1103/PhysRevLett.95.147201

[42] Suzuki, Y. et al. Propagation of avalanches in -acetate: Magnetic deflagration. *Phys. Rev. Lett.* **95**, 147201. 10.1103/PhysRevLett.95.147201 (2005). 16241690

[43] Charbonneau, P., McIntosh, S. W., Liu, H.-L. & Bogdan, T. J. Avalanche models for solar flares (Invited Review). *Solphys* **203**, 321–353 (2001).

[44] McLuckie, I. F. Investigation of zener and avalanche diode characteristics. *Physics Education* **23**, 251. 10.1088/0031-9120/23/4/417 (1988).

[45] Kohn, B. Particle Detection Using Magnetic Avalanches in Single-Molecule Magnet Crystals (2025) arXiv:2508.02467.

[46] Latino, G. et al. Quantum sensing for particle physics using Single Molecule Magnets. *Nucl. Instrum. Meth. A* **1079**, 170621 (2025).

[47] Miyazaki, Y. et al. Magnetic-field-dependent heat capacity of the single-molecule magnet [Mn12O12(O2CEt)16(H2O)3]. *Inorganic Chemistry* **40**, 6632–6636 (2001). 10.1021/ic010567w. 11735472

[48] Abe, K. et al. Search for Cosmic-Ray Boosted Sub-GeV Dark Matter Using Recoil Protons at Super-Kamiokande. *Phys. Rev. Lett.* **130**, 031802 (2023). [Erratum: Phys.Rev.Lett. 131, 159903 (2023)], arXiv:2209.14968. 10.1103/PhysRevLett.130.031802 36763398

[49] Granelli, A., Ullio, P. & Wang, J.-W. Blazar-boosted dark matter at Super-Kamiokande. *JCAP* **07**, 013 (2022) arXiv:2202.07598. 10.1103/PhysRevLett.128.221104 35714241

[50] An, H., Pospelov, M., Pradler, J. & Ritz, A. Direct detection constraints on dark photon dark matter. *Physics Letters B* **747**, 331–338. 10.1016/j.physletb.2015.06.018 (2015).

[51] Hochberg, Y., Pyle, M., Zhao, Y. & Zurek, K. M. Detecting superlight dark matter with fermi-degenerate materials. *Journal of High Energy Physics* **2016**, (2016). 10.1007/JHEP08(2016)057.

[52] Mitridate, A., Trickle, T., Zhang, Z. & Zurek, K. M. Dark matter absorption via electronic excitations. *JHEP* **09**, 123 (2021) arXiv:2106.12586.

[53] Catena, R. et al. Dark matter-electron interactions in materials beyond the dark photon model. *JCAP* **03**, 052 (2023) arXiv:2210.07305.

[54] Caputo, A., Millar, A. J., O’Hare, C. A. & Vitagliano, E. Dark photon limits: A handbook. *Physical Review D* **104** (2021). 10.1103/PhysRevD.104.095029.

[55] O’Hare, C. cajohare/axionlimits: Axionlimits. https://cajohare.github.io/AxionLimits/ (2020).

[56] Parker, S. R., Hartnett, J. G., Povey, R. G. & Tobar, M. E. Cryogenic resonant microwave cavity searches for hidden sector photons. *Phys. Rev. D* **88**, 112004 (2013) arXiv:1410.5244.

[57] Povey, R., Hartnett, J. & Tobar, M. Microwave cavity light shining through a wall optimization and experiment. *Phys. Rev. D* **82**, 052003 (2010) arXiv:1003.0964.

[58] Inada, T. et al. Results of a Search for Paraphotons with Intense X-ray Beams at SPring-8. *Phys. Lett. B* **722**, 301–304 (2013) arXiv:1301.6557.

[59] Kroff, D. & Malta, P. C. Constraining hidden photons via atomic force microscope measurements and the Plimpton-Lawton experiment. *Phys. Rev. D* **102**, 095015 (2020) arXiv:2008.02209.

[60] Arias, P. et al. WISPy Cold Dark Matter. *JCAP* **06**, 013 (2012) arXiv:1201.5902.

[61] McDermott, S. D. & Witte, S. J. Cosmological evolution of light dark photon dark matter. *Phys. Rev. D* **101**, 063030 (2020) arXiv:1911.05086.

[62] Witte, S. J., Rosauro-Alcaraz, S., McDermott, S. D. & Poulin, V. Dark photon dark matter in the presence of inhomogeneous structure. *JHEP* **06**, 132 (2020) arXiv:2003.13698.

[63] Caputo, A., Liu, H., Mishra-Sharma, S. & Ruderman, J. T. Modeling Dark Photon Oscillations in Our Inhomogeneous Universe. *Phys. Rev. D* **102**, 103533 (2020) arXiv:2004.06733. 10.1103/PhysRevLett.125.221303 33315459

[64] Dubovsky, S. & Hernández-Chifflet, G. Heating up the Galaxy with Hidden Photons. *JCAP* **12**, 054 (2015) arXiv:1509.00039.

[65] Wadekar, D. & Farrar, G. R. Gas-rich dwarf galaxies as a new probe of dark matter interactions with ordinary matter. *Phys. Rev. D* **103**, 123028 (2021) arXiv:1903.12190.

[66] Bhoonah, A., Bramante, J. & Song, N. Superradiant Searches for Dark Photons in Two Stage Atomic Transitions. *Phys. Rev. D* **101**, 055040 (2020) arXiv:1909.07387.

[67] Aralis, T. et al. Constraints on dark photons and axionlike particles from the SuperCDMS Soudan experiment. *Phys. Rev. D* **101**, 052008 (2020). [Erratum: Phys.Rev.D 103, 039901 (2021)], arXiv:1911.11905.

[68] Godfrey, B. et al. Search for dark photon dark matter: Dark E field radio pilot experiment. *Phys. Rev. D* **104**, 012013 (2021) arXiv:2101.02805.

[69] Nguyen, L. H., Lobanov, A. & Horns, D. First results from the WISPDMX radio frequency cavity searches for hidden photon dark matter. *JCAP* **10**, 014 (2019) arXiv:1907.12449.

[70] Ballou, R. et al. New exclusion limits on scalar and pseudoscalar axionlike particles from light shining through a wall. *Phys. Rev. D* **92**, 092002 (2015) arXiv:1506.08082.

[71] Ehret, K. et al. New ALPS Results on Hidden-Sector Lightweights. *Phys. Lett. B* **689**, 149–155 (2010) arXiv:1004.1313.

[72] Betz, M., Caspers, F., Gasior, M., Thumm, M. & Rieger, S. W. First results of the CERN Resonant Weakly Interacting sub-eV Particle Search (CROWS). *Phys. Rev. D* **88**, 075014 (2013) arXiv:1310.8098.

[73] Valle, Della & F. The PVLAS experiment: measuring vacuum magnetic birefringence and dichroism with a birefringent Fabry-Perot cavity. *Eur. Phys. J. C* **76**, 24 (2016) arXiv:1510.08052.

[74] Ayala, A., Domínguez, I., Giannotti, M., Mirizzi, A. & Straniero, O. Revisiting the bound on axion-photon coupling from Globular Clusters. *Phys. Rev. Lett.* **113**, 191302 (2014) arXiv:1406.6053. 25415896 10.1103/PhysRevLett.113.191302

[75] Dolan, M. J., Hiskens, F. J. & Volkas, R. R. Advancing globular cluster constraints on the axion-photon coupling. *JCAP* **10**, 096 (2022) arXiv:2207.03102.

[76] Andriamonje, S. et al. An Improved limit on the axion-photon coupling from the CAST experiment. *JCAP* **04**, 010 (2007) arXiv:hep-ex/0702006.

[77] Ajello, M. et al. Search for Spectral Irregularities due to Photon-Axionlike-Particle Oscillations with the Fermi Large Area Telescope. *Phys. Rev. Lett.* **116**, 161101 (2016) arXiv:1603.06978. 27152783 10.1103/PhysRevLett.116.161101

[78] Meyer, M., Giannotti, M., Mirizzi, A., Conrad, J. & Sánchez-Conde, M. A. Fermi Large Area Telescope as a Galactic Supernovae Axionscope. *Phys. Rev. Lett.* **118**, 011103 (2017) arXiv:1609.02350. 28106460 10.1103/PhysRevLett.118.011103

[79] Meyer, M. & Petrushevska, T. Search for Axionlike-Particle-Induced Prompt -Ray Emission from Extragalactic Core-Collapse Supernovae with the Large Area Telescope. *Phys. Rev. Lett.* **124**, 231101 (2020). [Erratum: Phys.Rev.Lett. 125, 119901 (2020)], arXiv:2006.06722. 10.1103/PhysRevLett.124.231101 32603156

[80] Davies, J., Meyer, M. & Cotter, G. Constraints on axionlike particles from a combined analysis of three flaring Fermi flat-spectrum radio quasars. *Phys. Rev. D* **107**, 083027 (2023) arXiv:2211.03414.

[81] Noordhuis, D. Novel Constraints on Axions Produced in Pulsar Polar-Cap Cascades. *Phys. Rev. Lett* **131**, 111004 (2023) arXiv:2209.09917. 37774289 10.1103/PhysRevLett.131.111004

[82] Dessert, C., Long, A. J. & Safdi, B. R. No Evidence for Axions from Chandra Observation of the Magnetic White Dwarf RE J0317–853. *Phys. Rev. Lett.* **128**, 071102 (2022) arXiv:2104.12772. 35244430 10.1103/PhysRevLett.128.071102

[83] Dessert, C., Dunsky, D. & Safdi, B. R. Upper limit on the axion-photon coupling from magnetic white dwarf polarization. *Phys. Rev. D* **105**, 103034 (2022) arXiv:2203.04319.

[84] Benabou, J. N. Search for Axions in Magnetic White Dwarf Polarization at Lick and Keck Observatories (2025) arXiv:2504.12377.

[85] Ning, O. & Safdi, B. R. Leading Axion-Photon Sensitivity with NuSTAR Observations of M82 and M87. *Phys. Rev. Lett.* **134**, 171003 (2025) arXiv:2404.14476. 40408723 10.1103/PhysRevLett.134.171003

[86] Janish, R. & Pinetti, E. Hunting Dark Matter Lines in the Infrared Background with the James Webb Space Telescope. *Phys. Rev. Lett.* **134**, 071002 (2025) arXiv:2310.15395. 40053972 10.1103/PhysRevLett.134.071002

[87] Pinetti, E. First constraints on QCD axion dark matter using James Webb Space Telescope observations (2025) arXiv:2503.11753.

[88] Saha, A. K., Bouri, S., Das, A., Dubey, A. & Laha, R. Shedding Infrared Light on QCD Axion and ALP Dark Matter with JWST (2025). arXiv:2503.14582.

[89] Todarello, E. et al. Robust bounds on ALP dark matter from dwarf spheroidal galaxies in the optical MUSE-Faint survey. *JCAP* **05**, 043 (2024) arXiv:2307.07403.

[90] Wang, H. Spectroscopic search for optical emission lines from dark matter decay. *Phys. Rev. D* **110**, 103007 (2024) arXiv:2311.05476.

[91] Nakayama, K. & Yin, W. Anisotropic cosmic optical background bound for decaying dark matter in light of the LORRI anomaly. *Phys. Rev. D* **106**, 103505 (2022) arXiv:2205.01079.

[92] Carenza, P., Lucente, G. & Vitagliano, E. Probing the blue axion with cosmic optical background anisotropies. *Phys. Rev. D* **107**, 083032 (2023) arXiv:2301.06560.

[93] Todarello, E. & Regis, M. Bounds on axions-like particles shining in the ultra-violet. *JCAP* **05**, 070 (2025) arXiv:2412.02543.

[94] Carosi, G. Search for Axion Dark Matter from 1.1 to 1.3 GHz with ADMX. *Phys. Rev. Lett* **135**, 191001 (2025) arXiv:2504.07279. 10.1103/d7mg-6sqq 41269976

[95] Wuensch, W. et al. Results of a Laboratory Search for Cosmic Axions and Other Weakly Coupled Light Particles. *Phys. Rev. D* **40**, 3153 (1989). 10.1103/physrevd.40.3153 10011684

[96] Bae, S. Search for Dark Matter Axions with Tunable TM020 Mode. *Phys. Rev. Lett* **133**, 211803 (2024) arXiv:2403.13390. 39642478 10.1103/PhysRevLett.133.211803

